# High efficient room temperature phosphorescent materials constructed with methylene molecular configuration

**DOI:** 10.3389/fchem.2022.1010676

**Published:** 2022-09-29

**Authors:** Jian Wang

**Affiliations:** School of Elementary Education, Chongqing Normal University, Chongqing, China

**Keywords:** room temperature phosphorescent, DFT, methylene, luminescence mechanism, nonradiative rates

## Abstract

In this work, we have investigated several pure organic room temperature phosphorescent materials with donor-methylene acceptor configurations with relatively different quantum efficiency. The results show that the introduction of methylene functional group in room temperature phosphorescent materials based on donor-acceptor configuration is more favorable for obtaining higher phosphorescent quantum efficiency in crystal phase environment. More importantly, our calculations reveal the root cause of the excellent quantum efficiency performance after the introduction of methylene groups. The results show that the introduction of methylene can inhibit the structural deformation of molecules during the excited state transition process and give them higher interaction. Moreover, in the donor-acceptor configuration, the heavy atom effect is more favorable to the formation of π-x (X = Br) interaction to accelerate the occurrence of intersystem crossing and achieve a higher intersystem crossing rate. Therefore, the donor-methylene-acceptor molecule is expected to improve the quantum efficiency of room temperature phosphorescence, and the addition of heavy atoms is more conducive to prolong the life of room temperature phosphorescence. This work provides a useful reference for rational design of room temperature phosphorescent materials with high efficiency and long life.

## Introduction

Room temperature phosphorescence (RTP) is a luminescent phenomenon different from fluorescence. It has attracted considerable interests due to their fundamental importance and potential applications in optoelectronics, sensors as well as bioimaging. (Baldo et al., 1998; Mi et al., 2010; You et al., 2011; Zhao et al., 2011) It is well known that fluorescent materials can only have a maximum exciton utilization rate of 25%.Therefore, people will turn their attention to phosphorescent materials with higher utilization rate. Early phosphorescent materials were composed of heavy metal complexes. (Ding et al., 2021; Liu et al., 2021; Zhang et al., 2021) Due to the disadvantages of heavy metal complexes such as high toxicity, high price and instability, more and more researchers turn to room-temperature phosphorescence (RTP) materials with low cost, molecular diversity, high energy utilization in excited state and long life. (Zhang et al., 2016; Li et al., 2017; Cai et al., 2019a; Cai et al., 2019b; Luo et al., 2019; Zhao et al., 2019; Zhao et al., 2020) However, achieving efficient room temperature phosphorescent emission in pure organic molecules is a huge challenge due to the instability of triplet exciton and the inefficient intersystem crossing (ISC) process caused by weak spin-orbit coupling (SOC). (Xiong et al., 2018; Lin et al., 2020; Ma et al., 2021).

The ideal high efficiency and long life RTP materials can be defined as:1) the molecular structure should have the ability to promote intersystem crossing from the single excited state to the triplet excited state, among which halogen and carbonyl groups are the most commonly used; 2) the molecule has an inherently rigid structure or is in a solid-state environment to reduce non-radioactive decay rates, inherently rigid structure usually means having conjugated aromatic rings; 3) Enhance the stability of triplet excitons through intermolecular coupling, face-to-face stacking structures with strong π-π interactions are generally considered to be one of the most favorable stacking methods. Compounds with carbonyl groups and conjugated aromatic rings are often used in room temperature phosphorescent materials because of their rigid structures that facilitate efficient intersystem crossing processes. For example, n-electron functional groups (such as aldehyde and carbonyl groups) are introduced in π -conjugated organic molecules to promote the intersystem crossing. (Pan et al., 2018; Wang et al., 2019; Li et al., 2020) These compounds have strong electron-coupled donor-acceptor skeletons, which tend to cause structural deformation during the excited state transition process, resulting in limited inhibition of molecular motion, or even inhibited by rigid environment. These rigid environments include polymerization, (Su et al., 2020; Wu et al., 2020) host-guest doping, (Lee et al., 2019; Zhang et al., 2019) self-assembly, (Gu et al., 2020; Zhang et al., 2020) crystallization, (Yang et al., 2020) etc. Therefore, the development of organic RTP with high efficiency and long life is a work worth exploring.

In the field of RTP materials, researchers have proposed a RTP material which is connected by methylene. Zhang et al. synthesized and characterized aggregation-induced emission with long-lived room temperature phosphorescence from methylene-linked organic donor–acceptor molecules. (Cai et al., 2018) Due to methylene, the molecule has a tetrahedron-like structure, which is conducive to the formation of a strong intermolecular interaction in the crystal phase, and can break the electron delocalization between donor and acceptor, and prevent the structural deformation of the excited state transition. In the previous work, we discussed room temperature phosphorescence of methylene linked organic donor-acceptor molecules synthesized and characterized by Zhang et al., under different environments. (Zhao et al., 2021).

Recently, Zhongfu An et al. achieved a phosphorescent quantum efficiency of 39.76% and a phosphorescent lifetime of 220.24 ms by introducing methylene and halogen functional groups into the donor-accepter structures to control structural deformation. (Yin et al., 2020) In addition, halogenated receptors can form intermolecular π-halogen interactions in crystals, accelerating ISC transitions without shortening RTP lifetimes. These experimental studies show that the introduction of methylene between donor and recipient has a profound effect on achieving efficient room temperature phosphorescence. However, how methylene regulate the properties of long-lived room temperature phosphorescence is not well understood.

**SCHEME 1:**
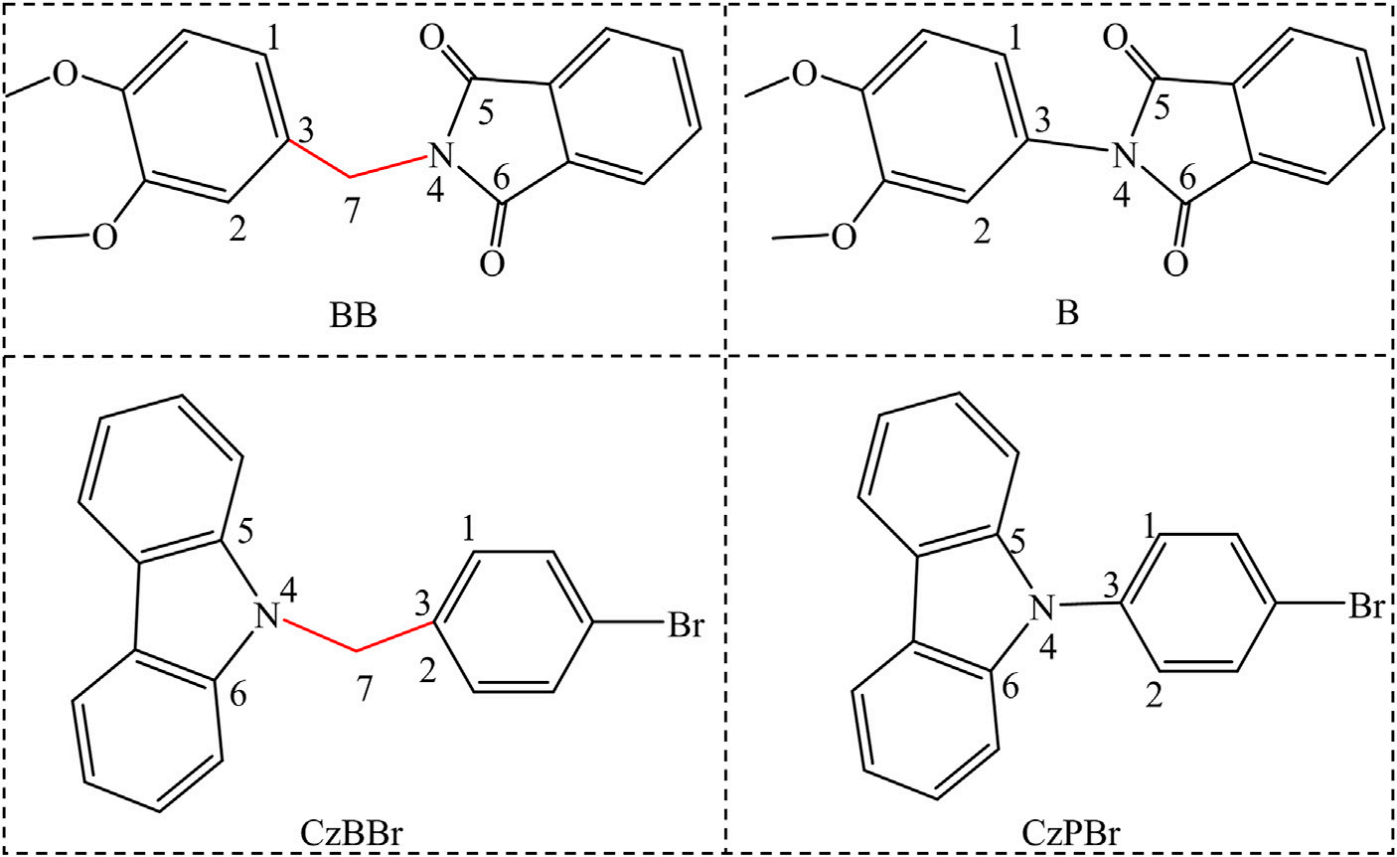
The structure of studied molecules.

To further understand the luminescence mechanism, based on BB and CzBBr molecules synthesized by An et al., we designed B molecules without methylene and CzPBr molecules containing halogens without methylene, as shown in Scheme 1. In this work, we use density functional theory to study the electronic structure properties of molecules, ONIOM model to simulate the crystal phase environment, and independent gradient model (IGM) method to analyze the interaction between molecules, hoping to clearly understand the essence of efficient room temperature phosphorescence. The radiative rate, non-radiative rate and intersystem crossing rate of all molecules were evaluated by quantitative calculation. We hope that these results can systematically elucidate the luminescence mechanism of donor-methylene-receptor or donor-methylene-halogenated receptor structures under solid phase conditions, so as to provide theoretical guidance for the preparation of efficient room temperature phosphorescent materials in experiments.

## Computational method

All molecules were structurally optimized at the ωB97XD/6–31 g ** level. Based on the optimized geometry, the frequencies of all molecules were calculated, and all frequencies had no virtual frequency to confirm that these structures were the lowest point in the potential energy surface. The crystal structure of molecule B were predicted by universal force field (UFF) by Polymorph module. (Rappe et al., 1993; Akkermans et al., 2013) We selected the five most probable space groups (P21/C, P2121, P1, P21, P1) and calculated by extracting the crystal structure of the space group with the lowest energy. By comparing the calculation results, we found that the space group of molecule B was P21/C, so we randomly selected one of them as the central molecule to construct ONIOM model. The crystal structures of other molecules were extracted from the Cambridge Crystal Database. The ONIOM model was used for QM/MM calculation, in which the central molecule was defined as the QM part and regarded as the high level, while the surrounding MM part was defined as the low level, and the molecules in the MM region were frozen during the frequency calculation. In the QM/MM calculation, the partial charge of atoms generated by restricted electrostatic potential (RESP) fitting method is used. The above calculation is obtained by using Gaussian 16. (Bredas et al., 2004; Frisch et al., 2016) The structural changes root-mean-square deviation (RMSD) of S0 and T1 states, S0 and S1 states and S1 and T1 states were calculated by VMD program. (Humphrey et al., 1996) The donor-acceptor dihedral Angle was obtained by Mercury program analysis. (Macrae et al., 2006) The spin orbit coupling (SOC) matrix elements between T1 and S0 states and between T1 and S1 are calculated using the quadratic response theory by Dalton 2016 program. (K et al., 2014; Vahtras et al., 1992) The Multiwfn program was used to calculate the natural transition orbits, and analyze the transition properties of molecules. (Lu and Chen, 2012) IGM method was used to visualize the interaction between molecules. (Lefebvre et al., 2017) Based on the data obtained above, MOMAP program was used to perform frequency analysis and calculate the intersystem crossing rate, radiative rate and non-radiative rate. (Niu et al., 2018).

## Results and discussion

### Structure of ground state and excited state

**FIGURE 1:**
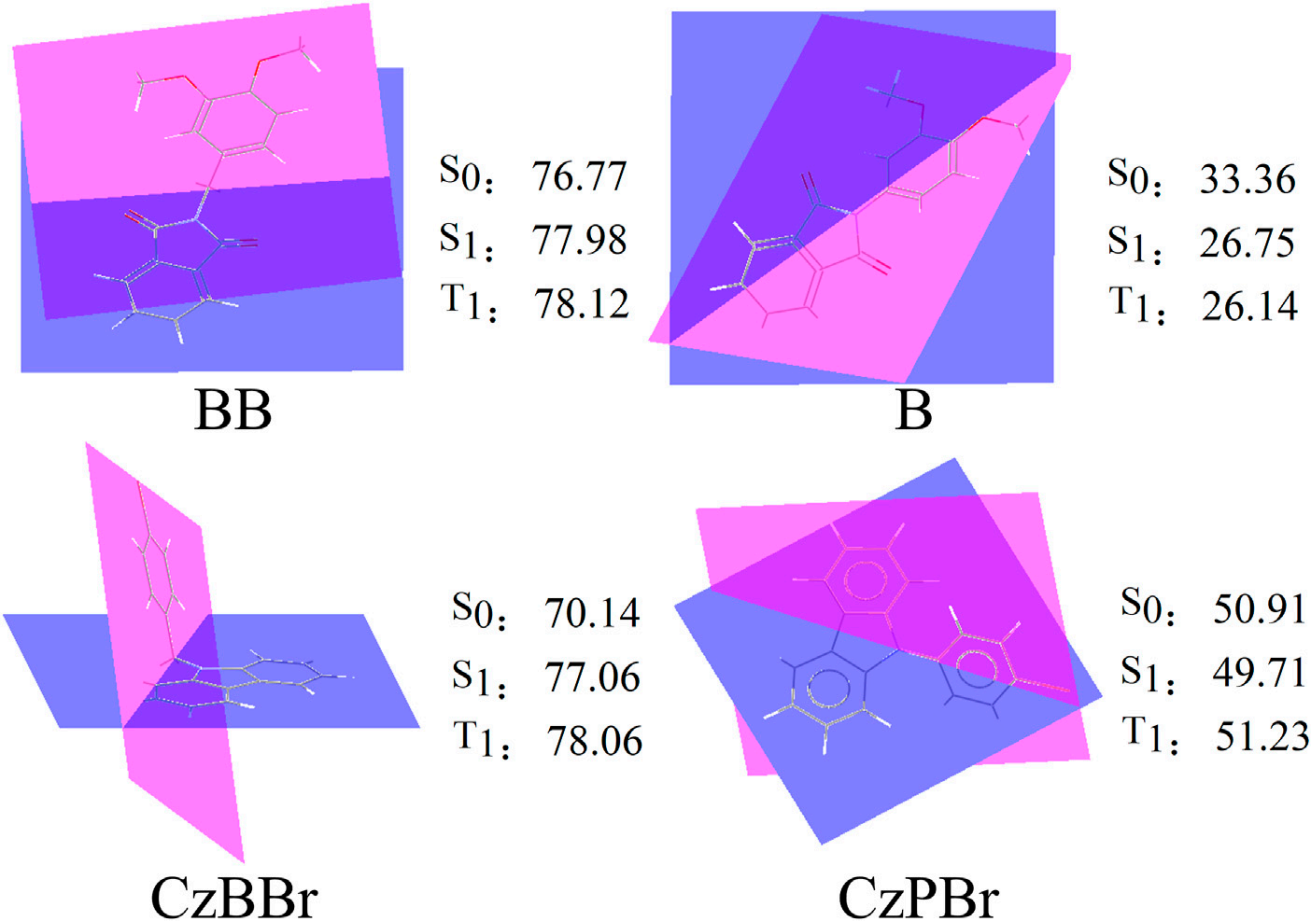
The dihedral Angle of studied molecules.

**FIGURE 2:**
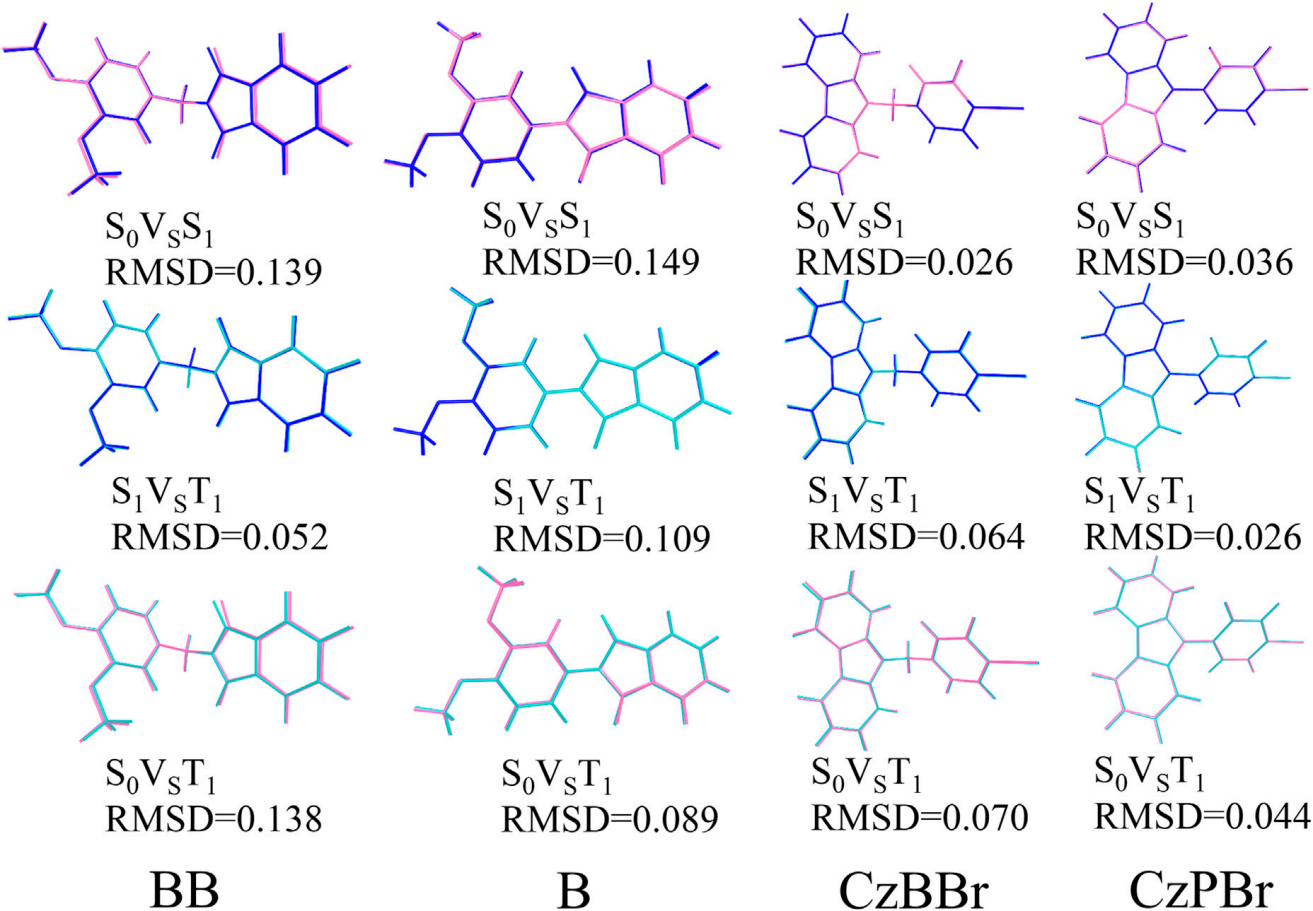
Comparison of S state and T state geometry, RMSD values of the studied molecules.

The geometrical and electronic structures of molecules play an important role in the photophysical properties, thus determining the luminescence efficiency of organic molecules. To investigate the effect of methylene and heavy atom effects on molecular structure, we calculated the dihedral Angle between donor and acceptor, as shown in Figure 1. In general, the dihedral Angle can reflect the degree of structural distortion of the material molecules. The dihedral angles of singlet S0 and first excited triplet T1 of BB are 76.77° and 78.12°, and the variation between them is less than that of molecule B (the dihedral angles of S0 and T1 states of molecule B are 33.36° and 26.14°). This indicates that the introduction of methylene is beneficial to inhibit molecular structural deformation. However, the dihedral angles of CzBBr and CzPBr are different. The dihedral angles of CzBBr in S0 and T1 states are 70.14° and 78.06°, and the dihedral angles of CzBBr in S0 and T1 states are 50.91° and 51.23°, respectively. The variation of dihedral Angle for S0 and S1 is consistent with that of S0 and T1. This indicates that the introduction of heavy atoms and methylene group has counteracting effect on the structural deformation. In order to quantitatively characterize the structural deformation during electron transition, we calculate the root mean square displacement (RMSD). $RMSD=\sqrt{\frac{1}{N}\overset{natom}{\underset{i}{\sum }}\left[{\left(X_{i}-X_{i}^{\prime }\right)}^{2}+{\left(Y_{i}-Y_{i}^{\prime }\right)}^{2}+{\left(Z_{i}-Z_{i}^{\prime }\right)}^{2}\right]}$ . The changes and RMSD values between S0, S1 and T1 states of all molecules are shown in Figure 2. RMSD values of S0 vs. S1, S1 vs. T1 and S0 vs. T1 of molecular BB were 0.139, 0.052, 0.138, respectively. The RMSD values of S0 vs. S1, S1 vs. T1 and S0 vs. T1 states of molecule B are 0.149, 0.109 and 0.089 respectively, indicating that the degree of structural distortion of molecule B is greater than that of molecule BB, which is consistent with the change of dihedral Angle discussed above. From the above analysis, it can be seen that the introduction of methylene and heavy atoms can cause geometrical changes of molecules, which may have a significant effect on the excited state properties of molecules.

### Energy level and transition properties

**FIGURE 3:**
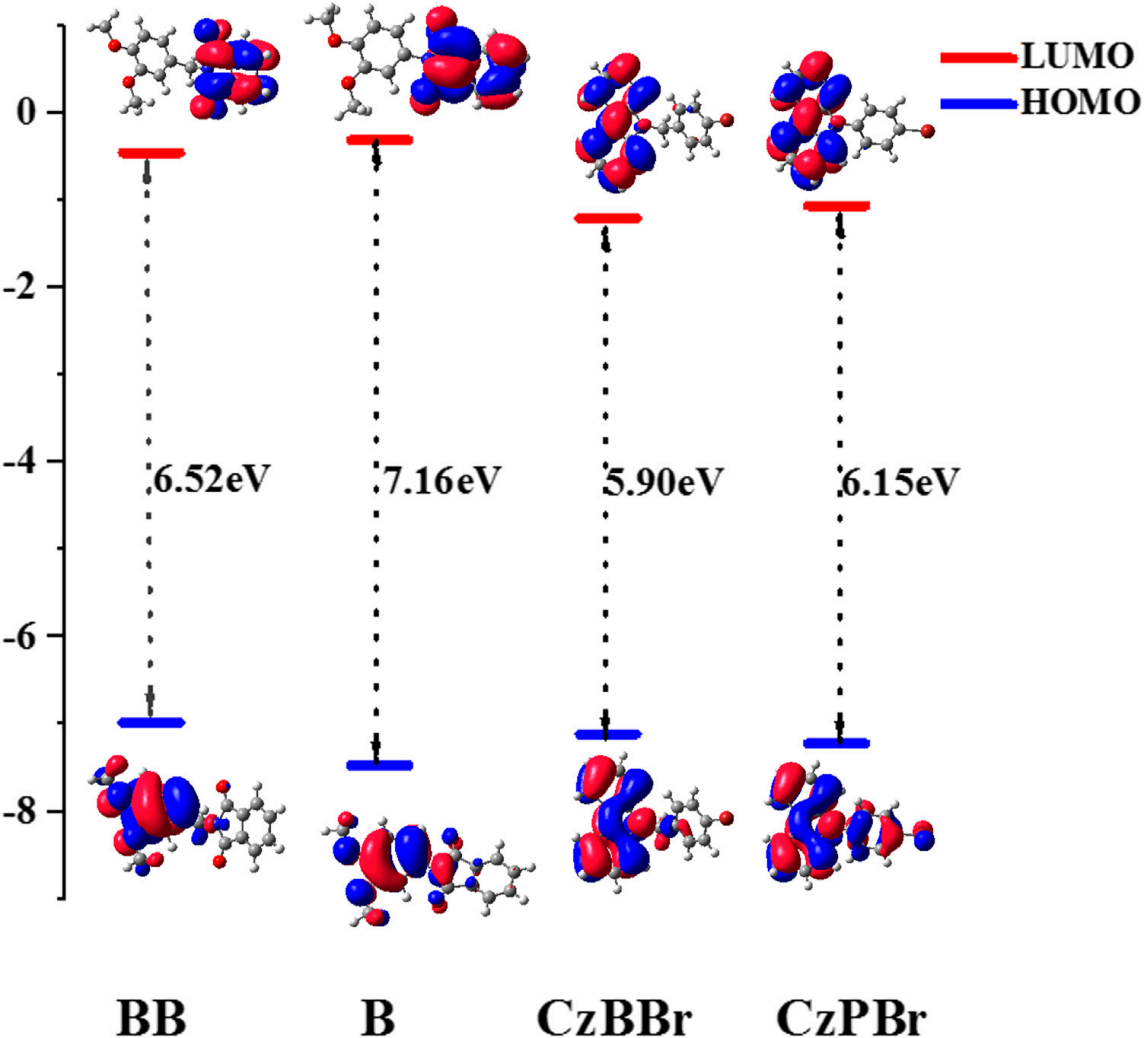
The HOMO and LUMO orbital diagram and energy gap of studied molecules.

To explore the electrical properties of molecules, the HOMO and LUMO levels of all molecules are calculated. As shown in Figure 3, the HOMO level of both BB and CzBBr molecules introduced with methylene is higher than that of B and CzPBr molecules without methylene. The results indicate that the introduction of methylene and heavy atom play an important role in the transition properties of molecules. It can be clearly seen from Figure 3 that the HOMO wave functions of BB and CzBBr molecules are mainly distributed on the donor, while the HOMO of B and CzPBr molecules are delocalized toward the acceptor. For LUMO, BB and B molecules are mainly distributed on the acceptor, while CzBBr and CzPBr molecules are mainly distributed on the donor unit.

**FIGURE 4:**
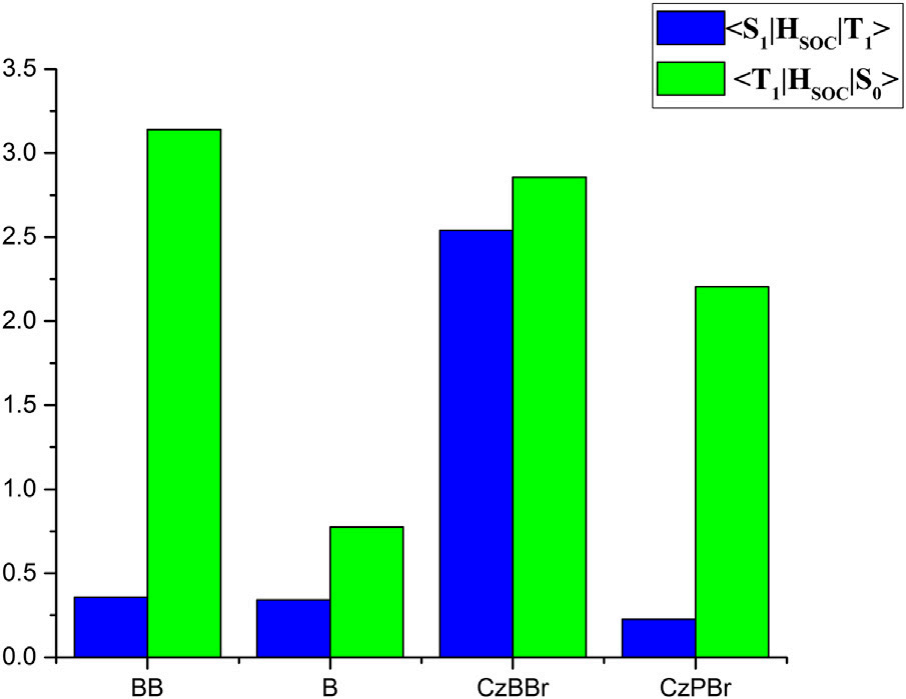
Schematic diagram of spin-orbit coupling constants.

**FIGURE 5:**
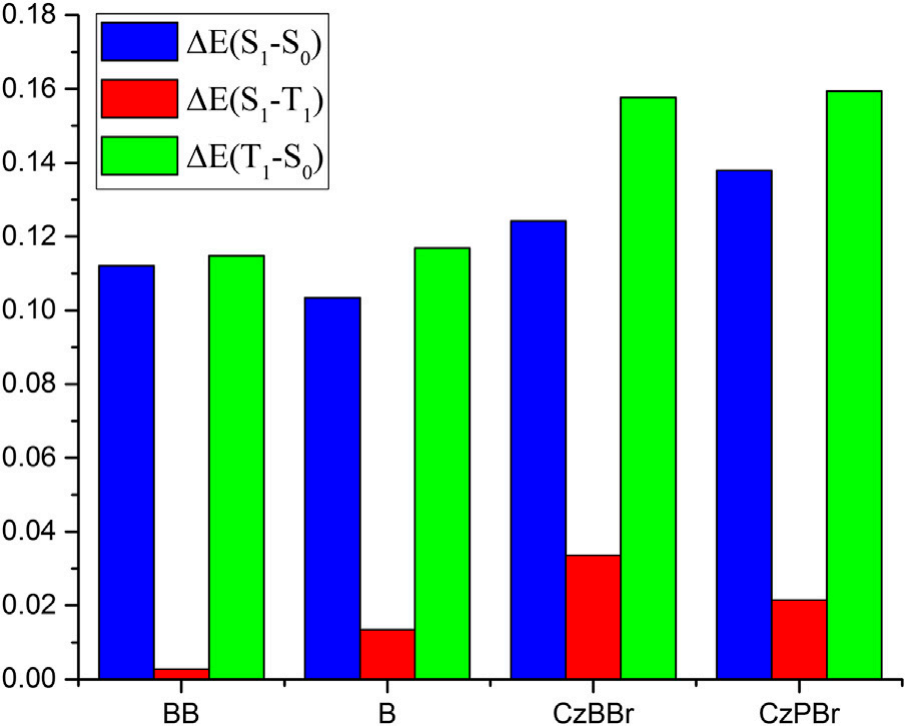
The comparison of adiabatic energy levels for different transition states.

The SOC between S1 and T1 states and between S0 and T1 states for studied molecules are illustrated in Figure 4. As shown in Figure 4 the SOC value between S1 and T1 states of CzBBr molecule (2.54 cm^−1^) was greater than CzPBr molecule (0.23 cm^−1^). For BB molecule and B molecule, there is little difference in SOC between them, which may be due to the absence of heavy atom effect. This indicates that CzBBr molecule has a faster ISC rate between S1 and T1 than CzPBr molecule, while BB and B molecule have similar ISC rates. Phosphorescent quantum efficiency and lifetime are two important parameters to evaluate the performance of RTP molecules. To calculate these two quantities, we optimized the excited states and calculated $\Delta E_{\left(S_{1}-T_{1}\right)}$ . As shown in Figure 5 and Table 1, the molecular BB, B, CzBBr and CzPBr were 0.07, 0.37, 0.91 and 0.59 eV, respectively.

**TABLE 1:** The excited state energy (eV) and energy gap (eV) of the studied molecule.

	BB	B	CzBBr	CzPBr
S_1_	3.12	3.18	4.29	4.34
T_1_	3.05	2.81	3.38	3.75
△E (S_1_ **-**T_1_)	0.07	0.37	0.91	0.59

**FIGURE 6:**
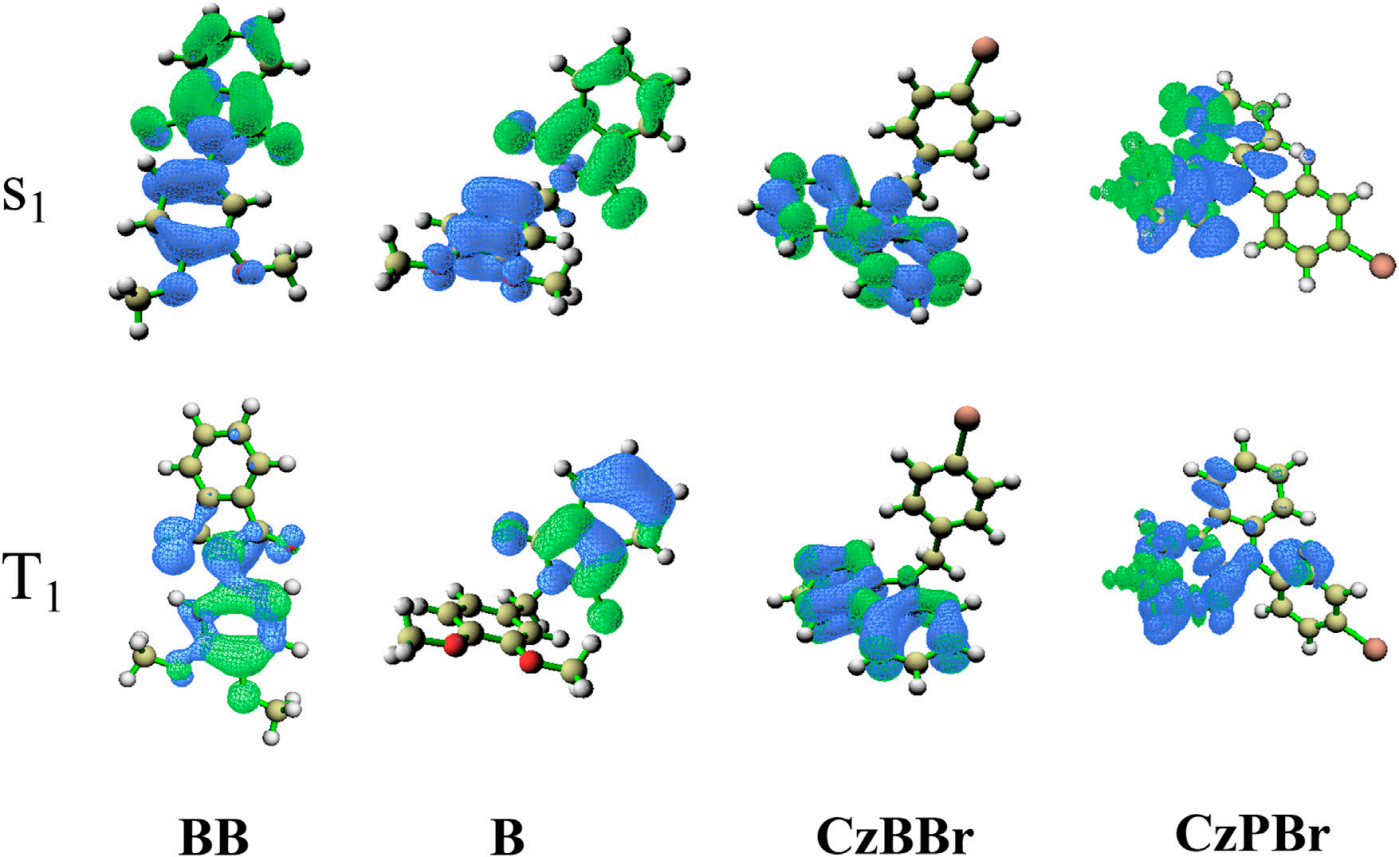
The distribution diagram of electron and hole.

It is worth noting that the energy difference of CzBBr molecule is the largest, which indicates that CzBBr molecule may have a small intersystem crossing rate, but this is contrary to the experimental value. The reason is that the SOC value between S1 and T1 states of CzBBr is large, which leads to a large intersystem channelage rate. This is consistent with the intersystem crossing rate calculated theoretically in the following paper. Similarly, the trend of $\Delta E_{\left(T_{1}-S_{0}\right)}$ is opposite to that of $\Delta E_{\left(S_{1}-T_{1}\right)}$ , which may be caused by the different oscillator strength of $T_{1}\to S_{0}$ (the oscillator strength of BB, B, CzBBr and CzPBr are 1.67 × 10^–8^, 1.74 × 10^–10^, 9.64 × 10^–11^, 6.76 × 10^–11^, respectively). In order to better explore the emission state properties, the electron hole distribution based on the first excited triplet state are calculated. As shown in Figure 6, compared with molecules B and CzPBr without methylene introduced, the T1 states of molecules BB and CzBBr are locally excited in the donor unit, while molecule B is locally excited in the acceptor unit, and the S1 states of molecules BB and B are charge transfer states distributed in the whole molecule. This shows that the nature of the excited states are changed along with the introduction of methylene and heavy atoms. Therefore, the introduction of methylene and heavy atoms is invaluable for the construction of efficient RTP.

### Radiative and nonradiative rates

In order to further explore the radiation situation, the calculated photophysical parameter of all molecules are listed in Table 2. As shown in Table 2, the intersystem crossing constants $k_{isc\left(S_{1}\to T_{1}\right)}$ of BB and CzBBr molecules with methylene group introduced are larger than those of B and CzPBr molecules without methylene group introduced, which is consistent with the orbital coupling and energy difference discussed above. In addition, the radiation rate constant (k_r_) of BB is increased from 1.66 to 52.6 s^−1^, and has the maximum value in selected molecules, due to the enhanced spin coupling value H_SOC_. It is noteworthy that the calculated phosphorescent quantum efficiency is in good agreement with the experimental value, which also proves the reliability of our calculation method.

**TABLE 2:** The optical physical property parameters of studied molecules.

	S_1_→T_1_	T_1_→S_0_		Φ_P_ (%)	
	k_isc_ (s^−1^)	K_r_ (s^−1^)	k_nr_ (s^−1^)	calcd	exptl
BB	1.87×108	52.6	1.20×103	4.2	3.5
B	1.32×105	1.66	9.07×103		
CzBBr	3.29×108	0.16	1.12×102		
CzPBr	8.87×104	0.26	0.51		

## Conclusion

In this work, we investigate two kinds of donor-methylene-acceptor and donor-acceptor pure organic room temperature phosphorescent materials with relatively different quantum efficiency. The results show that the introduction of methylene functional group in room temperature phosphorescent materials based on donor-acceptor configuration is more favorable for obtaining higher phosphorescent quantum efficiency in crystal phase environment. The results show that the introduction of methylene functional groups in room temperature phosphorescent materials can inhibit the molecular structural deformation during the excited state transition process, and can give it higher intermolecular interaction, which is more conducive to obtain higher phosphorescent quantum efficiency in crystal phase environment. In addition, the heavy atom effect is more favorable to the formation of π -x (X = Br) interaction in the donor-acceptor configuration, resulting in higher intersystem crossing rate. Therefore, the introduction of methylene and heavy atoms into donor-acceptor materials is expected to improve the quantum efficiency of room temperature phosphorescence and prolong the life of room temperature phosphorescence, which provides a new strategy for rational design of high efficiency and long life of room temperature phosphorescence materials.

## Data Availability

The original contributions presented in the study are included in the article/supplementary material, further inquiries can be directed to the corresponding author.
